# Mental health survey among front-line medical workers after 2 years of supporting COVID-19 efforts in Hubei Province

**DOI:** 10.1371/journal.pone.0287154

**Published:** 2023-10-17

**Authors:** Xianfeng Meng, Yan Wang, Yuna Jiang, Ting Li, Ying Duan

**Affiliations:** 1 The Mental Health Center of Liaoning Province, Shenyang, Liaoning, China; 2 Liaoning Delight Mental Health Service Company Ltd, Shenyang, Liaoning, China; 3 Panjin Kangning Hospital, Panjin, Liaoning, China; UV: Universiteti Ismail Qemali Vlore, ALBANIA

## Abstract

During the outbreak of COVID-19 in China, many health care workers have been involved in the front-line fight against the epidemic and have experienced major psychological challenges. This study was aimed at assessing the mental health of front-line health workers after 2 years of COVID-19 efforts. We recruited front-line health workers from Liaoning province who supported Hubei, the epicenter of the COVID-19 outbreak. The Patient Health Questionnaire-9 (PHQ-9), Generalized Anxiety Disorder scale (GAD-7), and Insomnia Severity Index (ISI) were used to assess psychological status. A total of 1101 of 1354 contacted individuals completed the survey (participation rate of 81.31%): 963 (87.5%) were 20–45 years of age, 919 (83.47%) were women, 845 (76.7%) were nurses, and 245 (22.3%) were physicians. After 2 years, the mental health symptoms among survey respondents were as follows: 46.6% had depression, 35.5% had anxiety, and 38.1% had insomnia. Thus, 2 years after the COVID-19 pandemic, the front-line health workers who had assisted Hubei province during the COVID‑19 pandemic in China still had high levels of depression, anxiety, and insomnia. Our findings suggest that the pandemic has had significant long-term effects on the mental health of front-line health workers. Therefore, mental health policies should offer long-term rather than short-term services.

## Introduction

A novel coronavirus, which the World Health Organization has called Coronavirus disease 2019 (COVID-19), was initially discovered in in Wuhan, Hubei Province, China [1]. This emerging infectious disease rapidly spread worldwide [2], and a total of 213 countries, areas, or territories were affected by April 2020 [3]. Globally, as of 4:16 pm CEST, 3 May 2023, 765,222,932 confirmed cases of COVID-19, including 6,921,614 deaths, had been reported to the WHO [4]. The most recent figures, before daily outbreak reporting from the Chinese government ceased, indicated 5,241 cumulative deaths and 397,195 confirmed cases [5]. During the COVID-19 pandemic, Shi et al. demonstrated that the prevalence rates of psychological symptoms in the general population in China were 27.9% for depression, 31.6% for anxiety, 29.2% for insomnia, and 24.4% for acute stress [6].

As of April 2020, 22,073 health care workers from 52 countries had been reported to be infected with COVID-19 by the WHO [7]. Front-line medical workers (FMWs) are exposed to high chronic stress because of their high risk of infection and long work hours. These constant stressors may negatively affect their sleep and mental health [8]. Furthermore, FMWs not only provide care for COVID-19 patients but also experience trauma, quarantine, social isolation, fears, and uncertainties [9]. Such high levels of stress, irregular work schedules, and frequent work shifts can lead to increased mental health problems [10]. A total of 78 Chinese healthcare workers died in the fight against COVID-19 between 23 January and 2 June 2020 [11], and an immense psychological burden was placed on the population, particularly among doctors and nurses, who were faced with high infection risks and increased workloads [12]. Approximately 42,000 medical workers, who were considered to be heroes in harm’s way, were dispatched to Wuhan City and Hubei Province from other parts of the country by the Chinese government to fight the COVID-19 pandemic, in the largest deployment of FMWs and medical resources worldwide [13]. Leishenshan and Huoshenshan Hospital are the two largest shelters in Wuhan, according to Wang et al. (2021). The COVID-19 pandemic affected FMWs’ resistance, self-reported sleep status, exhaustion, and anxiety levels at Wuhan Huoshenshan Hospital [14]. In one study, the psychological condition of the FMWs was monitored twice, after providing supporting work and spending 14 days in isolation, and high levels of anxiety and depressive symptoms remained present [15].

We conducted this study to investigate the mental health status of FMWs from Liaoning Province after 2 years of efforts in fighting against COVID-19 in Hubei.

## Materials and methods

### Study design

Self-reported questionnaires were used in an observational cross-sectional survey. The questionnaire was created on the Wenjuan Xing (www.wjx.cn) professional questionnaire survey network platform and then posted on the social media platform WeChat. The integrity check function of the platform was used to generate the online questionnaire; consequently, the questionnaire could not be submitted unless all questions were answered. To obtain more personal information, we compared the online questionnaire content with the name.

On the first page of the questionnaire, we presented an informed consent form, as shown in the supporting information (S1 File). Only participants providing informed consent could continue to answer the questionnaire. Thus the data for each participant in our study were obtained with informed consent. Our study was approved by the Ethics Committee of the Mental Health Center of Liaoning.

### Participants

The survey was conducted 2 years after the Wuhan outbreak. To recruit participants, we contacted the heads of each department and asked them to forward the questionnaire to their employees through WeChat. The participants in this survey were all medical staff in Liaoning Province who assisted Wuhan City and Xiangyang City in Hubei Province, which was the epicenter of the COVID-19 outbreak between February 2020 and April 2020. FWMs who worked in high-risk COVID-19 clinical departments, laboratories, and administrative departments were included in the study.

### Questionnaire

#### Socio-demographic characteristics

Information was collected on workers’ age (by year), gender (male or female), job title (junior, intermediate, or senior), marital status (married, unmarried, or divorced/widowed), education (college degree or below, bachelor’s degree, master’s degree, or doctoral degree), location where assistance was provided (Wuhan or Xiangyang), and occupation (physician, nurse, or public health staff). To further examine the association between mental health and related issues, we also gathered information with the following questions: "Were you satisfied with the welfare security after assistance project?" "Have you smoked in the last few months?" "Have you consumed alcohol in the last few months?" "Have you exercised weekly in the last few months?"

#### PHQ-9

Kroenke (2001) et al. developed the PHQ-9, a nine-item self-reported instrument for use in primary health care settings. This questionnaire can be used for tentative diagnosis and dimensional quantification of depression symptoms [16]. Each item represents a diagnostic criterion for major depressive episodes. Respondents indicate whether they experienced each symptom in the previous 2 weeks, with response options ranging from 0 to 3 (most days). The questionnaire can be completed in approximately 5–10 min. The reliability and construct validity of the PHQ-9 have been demonstrated, and older individuals were included in the validation samples [16,17]. The total PHQ-9 score was interpreted as normal (0–4), mild (5–9), moderate (10–14), or severe (15–28).

#### GAD-7

Generalized Anxiety Disorder-7 (GAD-7) is a short questionnaire that evaluates the level of anxiety during the previous 2 weeks [18]. It contains seven items rated on a 4-point scale (0–3), with a total score ranging from 0 to 21. Anxiety symptoms were defined by total scores of 5 or higher [19]. The GAD-7 has been found to be reliable and valid in Chinese studies [19]. Kroenke (2007) and Plummer (2016) have performed validation [20,21]. The total GAD-7 score was interpreted as normal (0–4) mild (5–9), moderate (10–14), or severe (15–28).

#### ISI

The presence of insomnia was measured with the Insomnia Severity Index (ISI), in which respondents rate each of the instrument’s seven items on a 5-point scale. The seven-item questionnaire is scored between 0 and 28 and has an acceptable internal consistency of 0.7 [22]. The ISI investigates difficulty in falling asleep, difficulty in remaining asleep, early morning awakenings, satisfaction derived from the sleep pattern, impairments emerging in daily functioning, awareness of sleep-associated impairments, and stress levels caused by sleep problems in the previous month. Research has indicated that the ISI is sensitive to detecting changes in patients’ perceptions of treatment outcomes, and a good degree of convergence exists between patients’ and the clinicians’ evaluations of insomnia severity [23]. The total ISI score was interpreted as normal (0–7), mild (8–14), moderate (15–21), or severe (22–28).

#### Statistical analysis

All statistical analyses were performed in IBM SPSS Statistics (version 26.0). The general data were described with descriptive analysis, and count data were analyzed with frequencies and percentages. We used a t-test and one-way ANOVA to compare the differences in related factors among the psychological status of FMWs, on the basis of the PHQ-9, GAD-7, and ISI. We used risk factor analysis to estimate potential factors affecting the mental health of FMWs. A corresponding 95% confidence interval (CI) was calculated, and the statistical significance level was set at P < 0.05.

## Results

### Sociodemographic characteristics of FMWs

In the study, we sent questionnaires to 1,338 FMWs, 1,101 (82.28%) of whom responded (Table 1). Almost all participants were front-line health care workers directly engaged in diagnosing, treating, or caring for patients who had, or were suspected to have, COVID-19; they had worked in Wuhan for 48.28 ± 7.88(M±SD) days and were isolated for 14.53 ± 2.94(M±SD) days after providing assistance.

**Table 1:** Socio-demographic characteristics of FWMs.

Variables	Number	Percentage(%)
**Total**	1101	100
**Gender**		
** Male**	182	16.5
** Female**	919	83.5
**Age, years**		
** 20–44**	963	87.5
** 45–65**	138	12.5
**Marital Status**		
** Married**	834	75.7
** Unmarried**	224	20.3
** Divorced/Widowed**	43	3.9
**Education**		
** College degree and below**	95	8.6
** Bachelor’s degree**	825	74.9
** Master’s degree**	137	12.4
** Doctor’s degree**	45	4.1
**Technical title**		
** Junior**	354	32.2
** Intermediate**	452	41.1
** Senior**	295	26.8
**Location of Assistance**		
** Wuhan**	910	82.7
** Xiangyang**	191	17.3
**Occupation**		
** Doctor**	245	22.3
** Nurse**	845	76.7
** Public health staff**	11	1.0
**Were you satisfied with the welfare security after assistance Project?**		
** Yes**	1014	92.1
** No**	87	7.9
**Have you smoked in the last months?**		
** Yes**	66	6.0
** No**	1035	94.0
**Have you consumed alcohol in the last months?**		
** Yes**	294	26.7
** No**	807	73.3
**Have you exercised weekly for the last months?**		
** Yes**	740	67.2
** No**	361	32.8

We added several questions measuring well-being and satisfaction after provision of assistance: smoking, alcohol consumption, and exercise (Table 1).

### Severity categories of depression, anxiety, insomnia symptoms, and prevalence

We assessed depression, anxiety, and insomnia as variables to examine the mental health of the FMWs at Wuhan and Xiangyang. The median (and IQR) scores of PHQ-9, GAD-7, and ISI among participants were 4.91 (95% CI:0.21–9.60), 3.32 (95% CI:0–7.18), and 5.0 (95% CI:1.12–11.81), respectively. Any symptoms overall in the participants, 46.6% (n = 503) scored 5 or higher on the PHQ-9, 33.5% (n = 369) scored 5 or higher on the GAD-7, and 38.1% (n = 419) scored 8 or higher on the ISI (Table 2).

**Table 2:** Symptom severity for depression, anxiety, and insomnia.

Variables	PHQ-9 N (%)				t/F	P	GAD-7 N (%)				t/F	P	ISI N (%)				t/F	P
	Normal	Mild	Moderate	Severe			Normal	Mild	Moderate	Severe			Normal	Mild	Moderate	Severe		
**Total**	588(53.4)	358(32.5)	114(10.4)	41(3.7)			732(66.5)	300(27.2)	54(4.9)	15(1.4)			682(61.9)	323(29.3)	83(7.5)	13(1.2)		
**Gender**																		
** Male**	90(49.5)	61(33.5)	19(10.4)	12(6.6)	4.494	0.315	112(61.5)	54(29.7)	14(7.7)	2(1.1)	4.783	0.155	581(63.2)	264(28.7)	65(7.1)	9(1.0)	1.676	0.07
** Female**	498(54.2)	297(32.3)	95(10.3)	29(3.2)			620(67.5)	246(26.8)	40(4.4)	13(1.4)			101(55.5)	59(32.4)	18(9.9)	4(2.2)		
**Age, years**																		
** 20–44**	507(52.6)	320(33.2)	102(10.6)	34(3.5)	1.228	0.449	637(66.1)	264(27.4)	52(5.4)	10(1.0)	1.198	0.937	596(61.9)	282(29.3)	75(7.8)	10(1.0)	0.732	0.851
** 45–65**	81(58.7)	38(27.5)	12(8.7)	7(5.1)			95(68.8)	36(26.1)	2(1.4)	5(3.6)			86(62.3)	41(29.7)	8(5.8)	3(2.2)		
**Marital Status**																		
** Married**	464(55.6)	263(31.5)	78(9.4)	29(3.5)	4.554	0.011*	557(66.8)	229(27.5)	34(4.1)	14(1.7)	1.584	0.206	521(62.5)	238(28.5)	63(7.6)	12(1.4)	1.455	0.455
** Unmarried**	106(47.3)	79(35.3)	29(12.9)	10(4.5)			150(67.0)	58(25.9)	16(7.1)	0(0.0)			138(61.6)	69(30.8)	16(7.1)	1(0.4)		
** Divorced/Widowed**	18(41.9)	16(37.2)	7(16.3)	2(4.7)			25(58.1)	13(30.2)	4(9.3)	1(2.3)			23(53.5)	16(37.2)	4(9.3)	0(0.0)		
**Education**																		
** College degree and below**	50(53.2)	30(31.9)	13(13.8)	1(1.1)	0.995	0.395	69(73.4)	19(20.2)	6(6.4)	0(0.0)	1.392	0.244	57(6.1)	28(29.8)	7(7.4)	2(2.1)	0.640	0.589
** Bachelor’s degree**	441(53.5)	263(31.9)	88(10.7)	33(4.0)			543(65.8)	230(27.9)	38(4.6)	14(1.7)			510(61.8)	242(29.3)	64(7.8)	9(1.1)		
** Master’s degree**	68(49.6)	54(39.4)	10(7.3)	5(3.6)			88(64.2)	41(29.9)	8(5.8)	0(0.0)			84(61.3)	42(30.7)	10(7.3)	1(0.7)		
** Doctor’s degree**	29(64.4)	11(24.4)	3(6.7)	2(4.4)			32(71.1)	10(22.2)	2(4.4)	1(2.2)			31(68.9)	11(24.4)	2(4.4)	1(2.2)		
**Technical title**																		
** Junior**	186(52.5)	112(31.6)	46(13.0)	10(2.8)	0.313	0.816	250(70.6)	82(23.2)	18(5.1)	4(1.1)	0.472	0.702	216(61.0)	110(31.1)	23(6.5)	5(1.4)	0.549	0.649
** Intermediate**	235(52.0)	161(35.6)	39(8.6)	17(3.8)			286(63.3)	137(30.3)	24(5.3)	5(1.1)			285(63.1)	130(28.8)	34(7.5)	3(0.7)		
** Senior**	167(56.6)	85(28.8)	29(9.8)	14(4.7)			196(66.4)	81(27.5)	12(4.1)	6(2.0)			181(61.4)	83(28.1)	26(8.8)	5(1.7)		
**Location of Assistance**																		
** Wuhan**	488(53.6)	296(32.5)	95(10.4)	32(3.5)	0.001	0.815	608(66.7)	249(27.3)	39(4.3)	15(1.6)	0.182	0.670	565(62.0)	265(29.1)	68(7.5)	13(1.4)	0.039	0.867
** Xiangyang**	100(52.6)	62(32.6)	19(10.0)	9(4.7)			124(65.3)	51(26.8)	15(7.9)	0(0.0)			117(61.6)	58(30.5)	15(7.9)	0(0.0)		
**Occupation**																		
** Doctor**	132(53.9)	77(31.4)	21(8.6)	15(6.1)	0.149	0.861	162(66.1)	67(27.3)	12(4.9)	4(1.6)	0.938	0.392	156(63.7)	67(27.3)	19(7.8)	3(1.2)	1.070	0.344
** Nurse**	453(53.6)	275(32.5)	91(10.8)	26(3.1)			567(67.1)	226(26.7)	41(4.9)	11(1.3)			521(61.7)	252(29.8)	62(7.3)	10(1.2)		
** Public health staff**	3(27.3)	6(54.5)	2(18.2)	0(0.0)			3(27.3)	7(63.6)	1(9.1)	0(0.0)			5(45.5)	4(36.4)	2(18.2)	0(0.0)		
**Smoking**					3.208	0.004**					4.437	0.005**					0.017	0.000***
** Yes**	22(33.3)	29(43.9)	9(13.6)	6(9.1)			34(51.5)	25(37.9)	5(7.6)	2(3.0)			26(39.4)	29(43.9)	9(13.6)	2(3.0)		
** No**	566(54.7)	329(31.8)	105(10.1)	35(3.4)			698(67.4)	275(26.6)	49(4.7)	13(1.3)			656(63.4)	294(28.4)	74(7.1)	11(1.1)		
**Drinking Alcohol**					3.556	0.000***					4.083	0.001**					1.936	0.001**
** Yes**	130(44.2)	100(34.0)	54(18.4)	10(3.4)			173(58.8)	94(32.0)	24(8.2)	3(1.0)			156(53.1)	105(35.7)	27(9.2)	6(2.0)		
** No**	458(56.8)	258(32.0)	60(7.4)	31(3.8)			559(69.3)	206(25.5)	30(3.7)	12(1.5)			526(65.2)	218(27.0)	56(6.9)	7(0.9)		
**Exercising**					3.988	0.000***					2.532	0.005**					0.030	0.001***
** Yes**	422(57)	230(31.1)	72(9.7)	16(2.2)			509(68.8)	187(25.3)	39(5.3)	5(0.7)			482(65.1)	199(26.9)	51(6.9)	8(1.1)		
** No**	166(46.0)	128(32.5)	42(11.6)	26(6.9)			223(61.8)	113(31.3)	15(4.2)	10(2.8)			200(55.4)	124(34.3)	32(8.9)	5(1.4)		
**Satisfied for welfare**					1.331	0.000***					5.793	0.000***					8.969	0.000***
** Yes**	560(55.2)	323(37.9)	96(9.5)	35(3.5)			685(67.6)	270(26.6)	48(4.7)	11(1.1)			644(63.5)	292(28.8)	69(6.8)	9(0.9)		
** No**	28(32.2)	35(40.2)	18(20.7)	6(6.9)			47(54.0)	30(34.5)	6(6.9)	4(4.6)			38(43.7)	31(35.6)	14(16.1)	4(4.6)		

Note: Abbreviations: PHQ-9, nine-item Patient Health Questionnaire (normal: 0–4; mild: 5–9; moderate: 10–14; severe: 15–28); GAD-7, seven-item Generalized Anxiety Disorder (normal: 0–4; mild: 5–9; moderate: 10–14; severe: 15–28); ISI, seven-item Insomnia Severity Index (normal: 0–7; mild: 8–14; moderate: 15–21; severe: 22–28). CI = confidence interval.

*0.05 > P-value > = 0.01.

**0.01 > P-value > = 0.001.

***P-value < 0.001.

**Fig 1:**
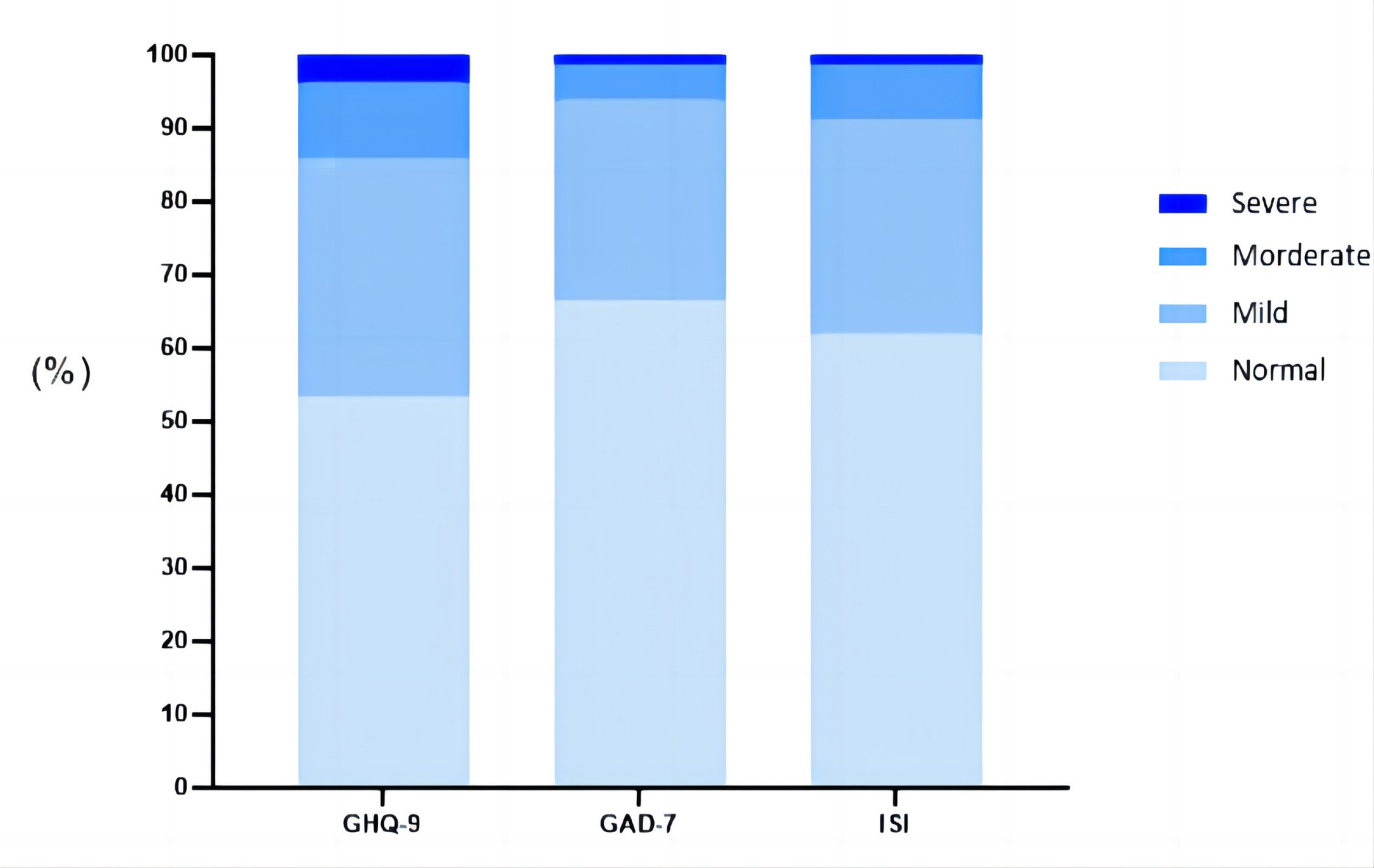
Distribution of the severity categories of depression, anxiety, and insomnia.

Given the clinical significance cut-off, the moderate and severe percentages were 14.1% for depression (n = 155; PHQ ≥10), 6.3% (n = 59) for anxiety, and 8.7% (n = 96) for insomnia (Fig 1). Among the 1101 participants, 169 (15.35%) had one or more moderate or severe symptoms. Of the 82 people with only one problem, the most common was depression (n = 71, 6.45%), and 11 (0.01%) had anxiety symptoms.A total of 53 (4.81%) had two symptoms, and 34 (3.09%) had symptoms of depression, anxiety, and insomnia.

Men had higher rates of moderate and severe depression (17.0%: 13.5%) and anxiety (8.8%: 5.8%), whereas women had higher rates of insomnia (12.1%: 8.1%). The younger group had higher rates of depression (14.1%:13.8%), anxiety (6.4%: 5.0%), and insomnia (8.8%: 8.0%), although no statistically significant difference was observed in scores (Table 2).

Marital status affected the incidence of mental disorders (F = 4.554, P = 0.011), and the scores for unmarried and divorced/widowed groups were significantly higher than those married on the depression (divorced/widowed, 21.0%; unmarried, 17.4%; married, 12.9%). Through the LSD test, the significance within the marital status group came from the significance between unmarried and married (F = 0.807, P = 0.022), married and divorced widows (F = −1.599, P = 0.029; Table 2).

In addition, we calculated scores and bio-demographic differences for depression, anxiety and insomnia with differing symptom severity (S1 Table).

### Risk factors associated with positivity rates of depression, anxiety, and insomnia

According to risk analysis, the smoking group had higher rates of moderate and severe depression (22.7%: 13.5%; OR = 2.599, P = 0.008), anxiety (10.6%: 6.0%; OR = 1.949, P = 0.001), and insomnia (16.6%: 8.2%; OR = 2.234; P = 0.000). Alcohol consumption was a risk factor in depression (21.8%: 11.2%; OR = 1.656; P = 0.02), anxiety (9.2%: 5.2%; OR = 1.577; P = 0.000), and insomnia (9.2%: 7.8%; OR = 1.494; P = 0.001). The non-exercise group had a higher percentage of moderate and severe conditions than the exercise group, according to the PHQ-9 (18.5%: 11.9%; OR = 1.559; P = 0.000), GAD-7 (7.0%: 6.0%; OR = 1.504; P = 0.001), and ISI (10.3%: 8.0%; OR = 1.318; P = 0.001). The prevalence of depression (27.6%: 13.0%; OR = 2.599; P = 0.000), anxiety (11.5%: 5.8%; OR = 1.772; P = 0.000), and sleeplessness (20.7%: 7.7%; OR = 3.130; P = 0.003) was higher among those who were dissatisfied with welfare after assistance(Table 3).

**Table 3:** Risk analysis of four factors in depression, anxiety, and insomnia.

Variables	PHQ-9(≥10)		GAD-7 (≥10)		ISI (≥15)	
	OR(95%CI)	P	OR(95%CI)	P	OR(95%CI)	P
**Total**						
**Smoking**						
** Yes**	2.599(1.630–4.144)	0.008*	1.949(1.900–2.000)	0.001**	2.234(1.304–3.83)	0.000***
** No**	Reference		Reference		Reference	
**Drinking Alcohol**						
** Yes**	1.656(1.634–1.678)	0.02*	1.577(1.476–1.682)	0.000***	1.494(1.291–1.730)	0.001**
** No**	Reference		Reference		Reference	
**Exercising**						
** Yes**	Reference		Reference		Reference	
** No**	1.559(1.210–2.008)	0.000***	1.504(1.163–1.944)	0.001*	1.318 (0.856–2.030)	0.001*
**Satisfied for welfare**						
** Yes**	Reference		Reference		Reference	
** No**	2.599(1.630–4.144)	0.000***	1.772(1.139–2.756)	0.000***	3.130(1.774–5.523)	0.003**

Note: Abbreviations: PHQ-9, nine-item Patient Health Questionnaire; GAD-7, seven-item Generalized Anxiety Disorder; ISI, seven-item Insomnia Severity Index. CI = confidence interval.

*0.05 > P-value > = 0.01.

**0.01 > P-value > = 0.001.

***P-value < 0.001.

## Discussion

On 31 December 2019, the World Health Organization reported an outbreak of COVID-19 in Wuhan, Hubei Province, China. An epidemic had never before had such a large and prolonged impact as the COVID-19 pandemic, which has posed substantial psychological challenges among healthcare workers (e.g., high risk of infection, physical exhaustion, and effects on mental health due to loss of the infected patients, personal safety, and fear of passing infections to family members) [24]. The effects of the pandemic on the mental health of people working at the front-lines in COVID-19 patient treatment, caretakers, and healthcare personnel has been a topic of interest [25]. Several studies have investigated the psychological status of health care workers [26–30], the general population [6], and public health workers [31] during the outbreak period.

To our knowledge, this study is the first to examine the mental health status of FMWs after they had supported pandemic efforts in Hubei Province for 2 years. This study’s results indicated that the combined prevalence of having at least one mental disorder was as high as 55.86% (mild above, n = 615), a percentage higher than the 38% previously reported [32].

A total of 46.6% of FMWs had mild or moderate depression symptoms, and several studies have reported percentages of 31.6% [15] to 50.7% [28]. The prevalence of depression symptoms was higher than that in the general population (27.9%) [6]. The above studies used the same tool (PHQ-9). Two studies calculated the average score, and our results of 4.91 (95% CI:0.21–9.60) were higher than the previously reported 4.0 (95% CI:2.0–8.0) [29], but lower than the previously reported 5.59 (95% CI:0.45–10.63) during the outbreak period and 4.67 (95% CI:0.40–8.94) during the stable phase among nurses [33].

A total of 35.5% of FMWs had mild or moderate anxiety symptoms, similarly to the 35.4% [34] reported in a study conducted 3 months after the COVID-19 outbreak. These proportions were higher among medical staff than the general population (31.6%) [6]. A total of 39.3%–51.4% of FMWs had anxiety symptoms [28,29,34,35], on the GAD-7 scores were higher than our study 35.5%, 23.6% [15] and 31.6% [6]. Our study calculated an average score of 3.32 (95% CI:0–7.18), which was higher than the previously reported 3.0 (95% CI:0.0–7.0) [29] during the outbreak period and lower than the 3.61 (95% CI:0.20–7.07) during the stable phase in nurses [33], on the basis of the GAD-7.

Additionally, 38.1% of FMWs had poor sleep quality; this percentage was higher than the previously reported 19.7% (26% for staff from Wuhan; 10.3% for staff from outside Wuhan) [32] and lower than the 45.5% [8] reported in studies using a different scale (Pittsburgh Sleep Quality Index). One study used the same measure but evaluated only nurses during the COVID-19 outbreak, and reported 38.5% in the outbreak phase and 39.9% in the stable phase [33]. These results were all higher than the 24.4% observed in the general population [6].

Given the clinical importance of these symptoms, the score in our study was 14.15% for moderate or higher depression; these percentages were lower than the 17.3% [29] and 14.8% [35] reported in the other two similar studies. The prevalence rate of nurses with more than moderate symptoms was consistent with the prevalence rate of anxiety symptoms in Cai’s study [33], but the overall prevalence rate of anxiety symptoms with more than moderate symptoms was lower than that in Chen’s study 12.3% [35], and the prevalence of symptoms of insomnia with more than moderate symptoms was higher than that in Chen’s study 7.8% [35].

Our results indicated higher comorbidities of depression, anxiety, and insomnia. Yue et al. have indicated that the sleep quality among FMS with anxiety and depression is poorer than that in FMS with only depression [27]. Patients with both depression and anxiety symptoms have been found to have a greater frequency of sleep difficulties [36]. Several reviews and meta-analyses on related studies, mostly from within China, have indicated a lower prevalence of depression, anxiety, and insomnia than that observed in our study [37–39]. A review has illustrated that the risk of mental disorders in the COVID-19 outbreak was associated with occupational factors (FMWs’ direct contact with COVID-19, availability of personal protective equipment (PPE), and heavy workload), psychosocial factors (fear of infection and concerns regarding family), sociodemographic factors (younger age, being female, having underlying illness, or being an only child), environmental factors (point in the pandemic curve, geography, and protective factors against adverse mental health outcomes) [37]. Although we did not know the current prevalence rate in the general population or in the population working in health care when the participants were assessed 2 years after providing support at the epicenter, high levels were nonetheless observed. Previous studies have suggested that medical workers are particularly vulnerable to mental health problems even during times of a relative epidemic decline [40,41]. Other results have suggested that stress and fatigue among front-line health workers may be associated with the risk of adverse mental health outcomes [14,26,34]. Our results are largely consistent with those of other studies, which have reported that, during major public health emergencies, medical staff face a risk of experiencing serious mental health consequences due to high-intensity fatigue. However, most healthcare professionals investigated herein were almost out of the pandemic’s path, and we did not know whether this was due to the epidemic or other factors. A study among front-line nurses in the Philippines during an outbreak has found that social support, personal resilience, and organizational support all influenced their anxiety [42].

Assessment of the bio-demographics indicated that unmarried, divorced, or widowed participants had significantly higher depression scores than married participants. Similar results have been found in the general population of Nigeria during the COVID-19 lockdown [43]. This finding contrasted with other results showing differences in lower annual household income, family members or relatives with suspected or confirmed SARS-CoV-2 infection, comorbidity, deteriorating relationships with family members [34], age (31–40 year group), educational background, and appraisal of the threat of infection by the virus [44]. We also analyzed several key influencing factors and found that smoking, drinking alcohol, exercising, and being satisfied with welfare all substantially influenced mental status. Study findings have revealed that exercise was associated with depressive symptoms in the initial phase of the lockdown, thus indicating that exercise may protect against stress-induced depression, but severe stress may negate this benefit [34]. The dissatisfaction with welfare among participants might have been because the government or hospital did not hire them as permanent employees, i.e., those with long-term, stable jobs, and equal pay for equal work by other permanent employees. Therefore, to avoid such factors, we can consider when sending FMWs who were permanent employees to the epicenter.

This study has substantial public mental health significance in the context of the novel coronavirus pandemic. First, this study is similar to many others demonstrating the effects of COVID-19 on mental health [45,46], particularly among FMWs [32,47,48]. Second, in comparison to the above psychological surveys of FMWs in China, we found high prevalence of psychological symptoms after 2 years of work at the front-lines of the epidemic. Third, reviews have suggested public mental health strategies to address the psychological problems associated with the pandemic[49–51], but the policies regarding mental services associated with the pandemic have often been temporary or interim, or associated with specific events, such as when an organization is engaged in supporting anti-epidemic activities, or when services are enhanced in response to a local outbreak but then suddenly disappear. Although some studies have indicated that psychological problems peak after 1 year [52], our study demonstrated high levels of psychological symptoms after 2 years, thus suggesting that psychological service policies should be sustained and extended. Finally, and most importantly, very little is known about the utilization of mental health services by FMWs. If utilization is absent or scant, barriers may exist to service use, such as stigma or perceived need. These aspects will serve as inspiration for future research.

## Limitations

This investigation has several limitations that must be considered in the interpretation of our results. First, this was a cross-sectional study. Therefore, control group data collection was not performed (e.g., non-supported front-line medical staff and general populations). We cannot infer causality in the interpretation of the outcomes. Second, because the tools used were meant for preliminary screening of the presence of psychiatric conditions, but they are not indicative of a clinical diagnosis.

## Conclusions

After 2 years of supporting the epidemic center in Hubei Province, FMWs still had high rates of anxiety, depression and insomnia symptoms. Additionally, regular exercise is a protective factor, whereas smoking, drinking alcohol, and dissatisfaction with welfare benefits were risk factors for mental health issues. When FMWs are sent to provide assistance after an epidemic outbreak, these factors should be considered. Additionally, COVID-19 has long-term effects on mental health; therefore, corresponding mental health policies should offer long-term rather than short-term services.

## Supporting information

S1 Table The Scores of symptoms severity of for depression, anxiety, and insomnia. (XLSX)

S1 File The presentation of informed consent for participation in the investigation. (DOCX)
