# Supplementary material for: Mental health survey among front-line medical workers after 2 years of supporting COVID-19 efforts in Hubei Province
Source: PLoS One. 2023 Oct 17;18(10):e0287154. doi: 10.1371/journal.pone.0287154 (PMC10581499; doi:10.1371/journal.pone.0287154)
Supplement: S1 File — (DOCX) [file pone.0287154.s002.docx]

**S1 File. Informed consent for participation in the investigation (for review purposes only)**


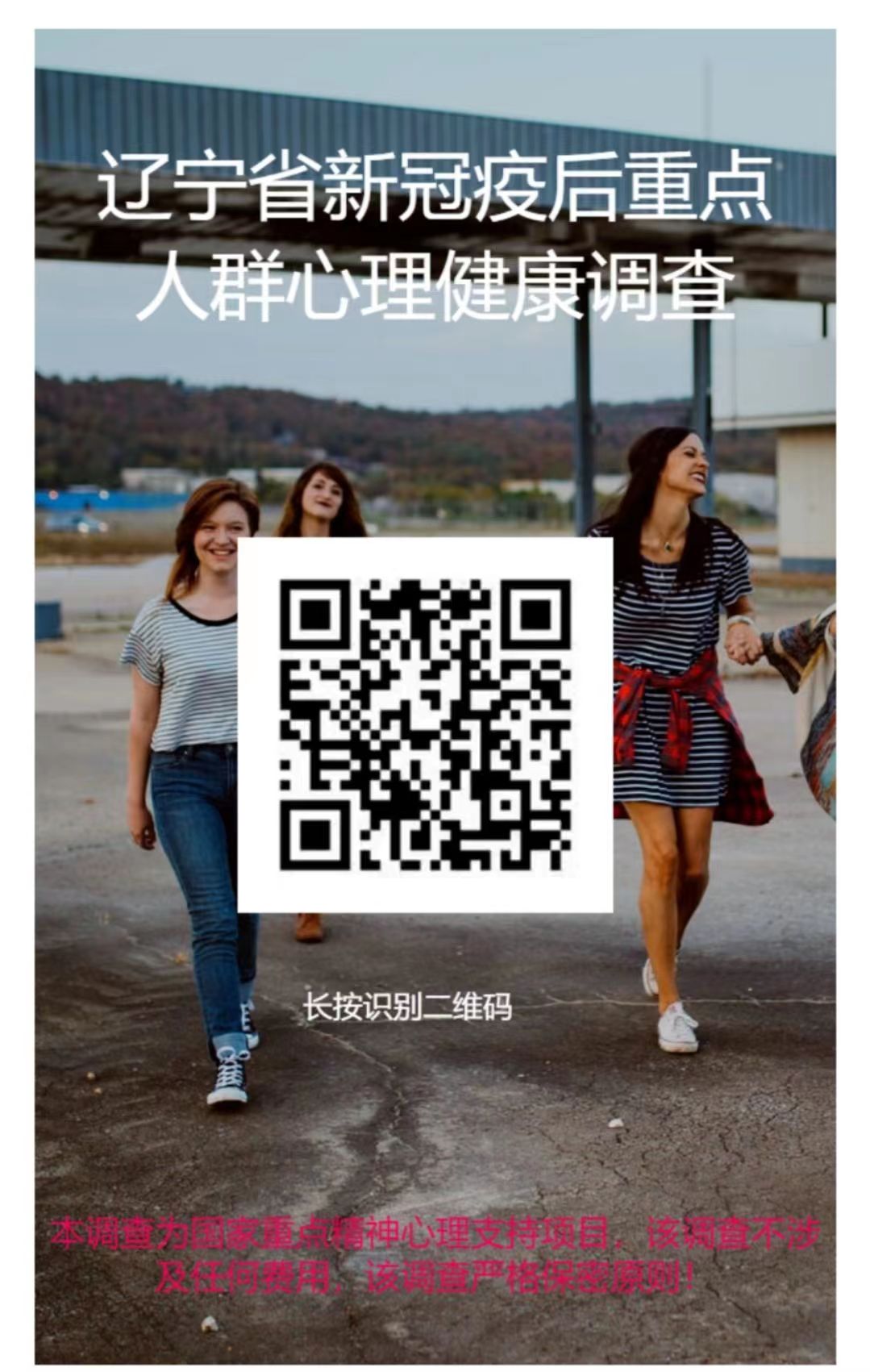


**Mental health survey of key population after COVID-19 in Liaoning Province**

**Online surveys accessed through QR codes**


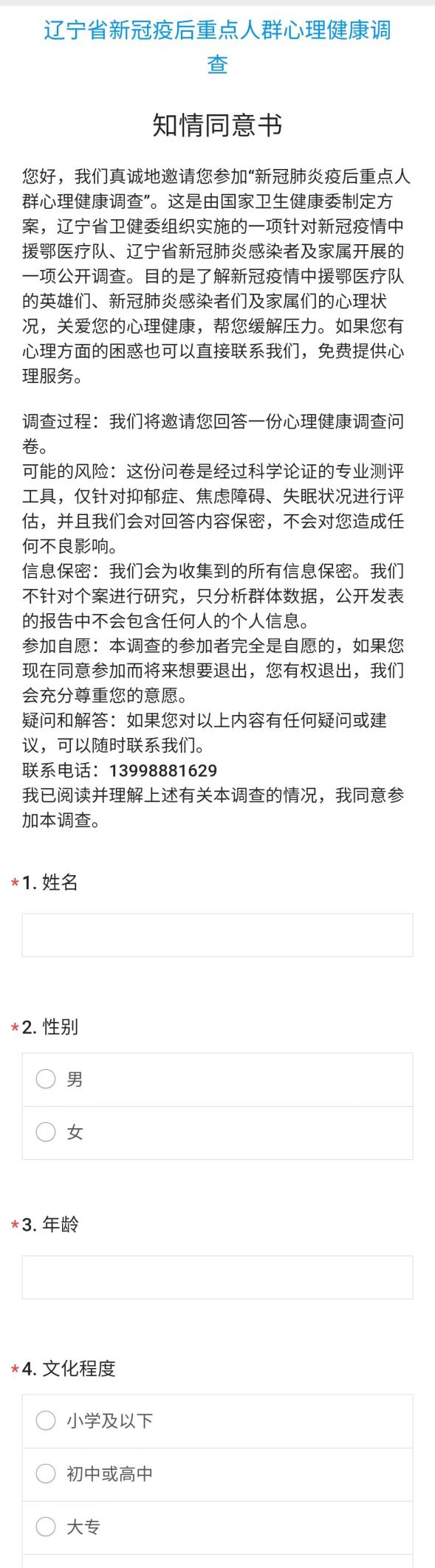


**Informed consent**

Hello, we sincerely invite you to participate in the "Mental health Survey of key population of COVID-19". Formulated by the National Health Commission and organized by the Liaoning Provincial Health Commission, this is a public investigation into the medical teams that assisted Hubei in the COVID-19 outbreak, as well as people infected with COVID-19 in Liaoning and their families. The purpose is to understand the psychological conditions of the heroes of the medical team in Hubei, those infected with COVID-19 and their families, care for your mental health, and help you relieve pressure. If you have psychological confusion can also contact us directly, free psychological services.

**Survey process:** You will be invited to answer a mental health questionnaire.

**Possible risks:** This questionnaire is a scientifically proven professional assessment tool, only for depression, anxiety disorders, insomnia assessment, and we will keep the answers confidential, will not cause any harm to you.

**Confidentiality:** All information collected will be kept confidential. We do not study individual cases. We only analyze population data, and our published reports do not contain anyone's personal information.

**Voluntary participation:** Participants in this survey are completely voluntary. If you agree to participate now and want to withdraw in the future, you have the right to withdraw. We will fully respect your wishes.

**Questions and Answers:** If you have any questions or suggestions about the above, please write them in the line below. Feel free to contact us in the future.

Contact number: +8613998881629

I have read and understood the above information about this survey, and I agree to participate in this survey.

Agree and continue to answer

**English translation of informed consent**
